# A Compact Prism‐Based Microscope for Highly Sensitive Measurements in Fluid Biopsy

**DOI:** 10.1002/jbio.202400519

**Published:** 2025-02-05

**Authors:** Laura Perego, Caterina Dallari, Chiara Falciani, Alessandro Pini, Lucia Gardini, Caterina Credi, Francesco Saverio Pavone

**Affiliations:** ^1^ Department of Physics University of Florence Sesto Fiorentino Italy; ^2^ European Laboratory for Non‐Linear Spectroscopy (LENS) University of Florence Sesto Fiorentino Italy; ^3^ National Institute of Optics (INO) National Research Council (CNR) Sesto Fiorentino Italy; ^4^ Department of Medical Biotechnology University of Siena Siena Italy; ^5^ SetLance Srl Siena Italy; ^6^ Clinical Pathology Unit Santa Maria Alle Scotte Hospital Siena Italy

**Keywords:** biosensors, compact set‐up, lipopolysaccharides, nanoparticles, prism‐based evanescent wave, scattering

## Abstract

The increasing demand for sensitive, portable, and affordable disease detection methods has spurred the development of advanced biosensors for rapid early‐stage diagnosis, population mass screening, and bed‐monitoring. Current high‐sensitivity devices face hurdles such as high production costs and challenges in multiplexed signal detection. To address these, we developed a prism‐based total internal reflection system which, in combination with surface functionalization techniques of gold nanoparticles, enables evanescent wave scattering for highly sensitive and rapid detection of specific analytes in both synthetic and human liquid samples. To validate its efficacy, we conducted scattering experiments in synthetic and human serum samples, exploiting functionalized AuNPs to recognize bacterial lipopolysaccharides as biomarkers for sepsis disease. We demonstrate a remarkable sensitivity in the femtogram per mL concentration range for this specific pathological biomarker. Based on this result we envisage the potential adoption of our technique for liquid biopsy in the clinical scenario.

## Graphical abstract

The growing need for portable, affordable disease detection has led to the development of advanced biosensors. We created a prism‐based total internal reflection system with gold nanoparticles to enable highly sensitive, rapid detection of analytes. Using this system, we detected bacterial lipopolysaccharides in synthetic and human serum as sepsis biomarkers with remarkable sensitivity in the femtogram per mL range, suggesting potential for liquid biopsy in clinical settings.

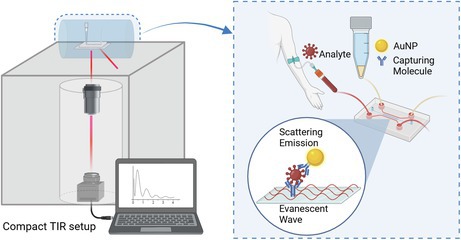

## 1 Introduction

**FIGURE 1:**
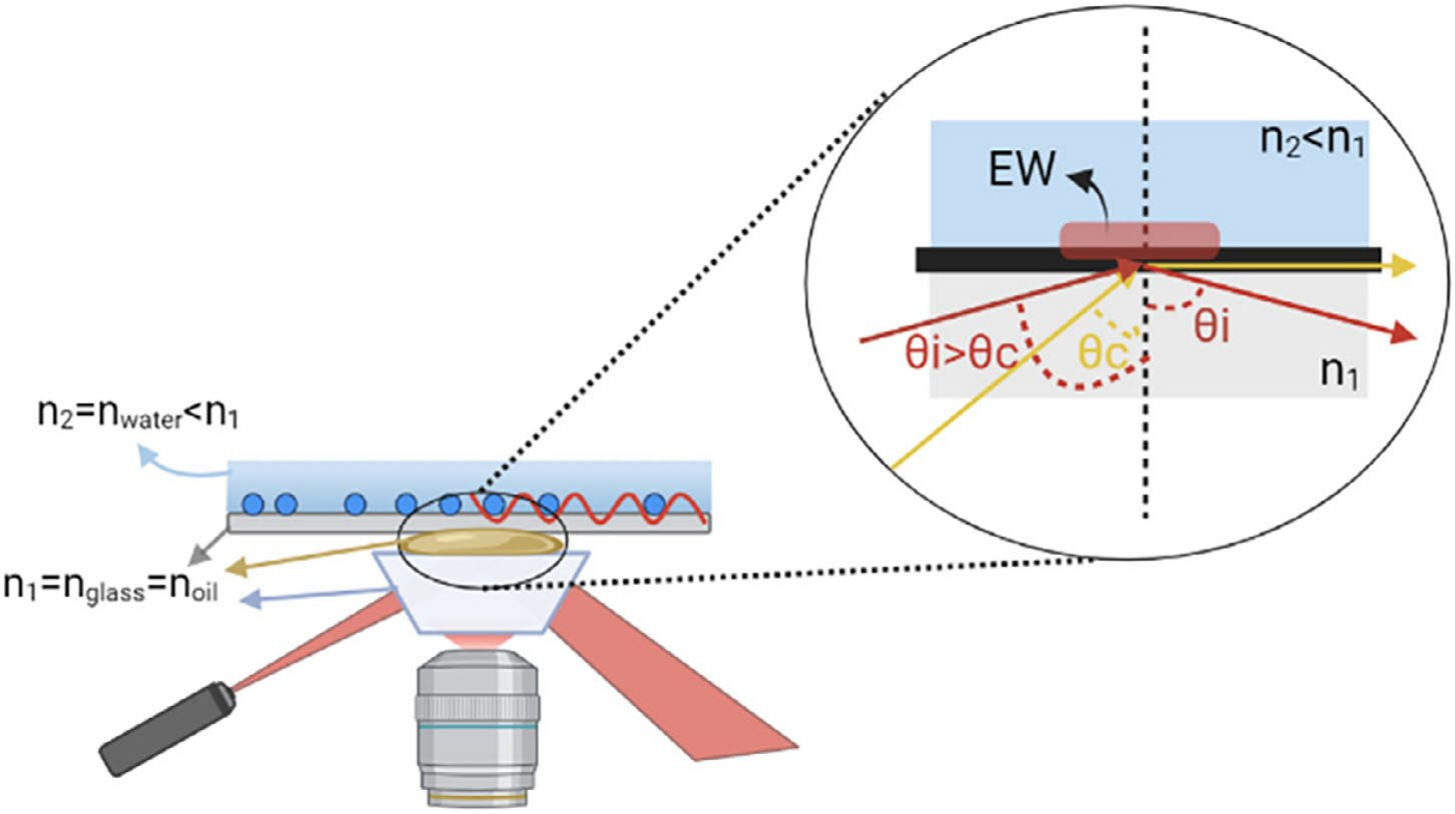
Graphical scheme of the optical coupling between the glass microprism and the glass coverslip through immersion oil, with the same refractive index of glass. A refractive index mismatch occurs at the interface between the glass coverslip and the sample buffer. As a consequence, total internal reflection occurs at this interface, generating an EW, which propagates within the sample. The inset shows the optical path of light rays at the interface between the two media, following the Snell's law.

In recent years, liquid biopsy has emerged in the clinical scenario as a powerful, straightforward, and non‐invasive diagnostic alternative to “gold‐standard” assays and tissue biopsy, such as histological analysis or genetic evaluation [1]. However, standard biopsy methods present several disadvantages, being highly invasive, risky, and impractical, especially for deep‐seated lesions. These methods may also require lengthy and costly analytical procedures and specialized equipment. The emergence of liquid biopsy as an alternative to traditional biopsy techniques raises the need for novel technologies. Thus, significant efforts have been directed toward the development of new highly‐ sensitive technologies enabling the analysis of body fluids and the detection of abnormal concentrations or alterations of pathological biomarkers with improved sensitivity and specificity [2]. Recurrent monitoring of biomarkers could provide real‐time insights into a patient's status, supporting both the prevention screening, before the disease progression, and the treatment decisions by following the pathology's dynamics [3, 4]. In this scenario, total internal reflection (TIR) spectroscopy has been exploited as a non‐destructive, selective, and extremely sensitive method to analyze adsorption phenomena and biomolecular interactions in vitro [5]. In particular, TIR microscopy exploits the reflection phenomenon at the interface between two materials with different refractive indices. As shown in Figure 1 (inset), when a collimated beam impacts the interface between a glass coverslip and the water in the sample solution at an angle of incidence greater than the critical angle, TIR occurs, following Snell's law, and the incident beam is reflected back from the glass surface along the same optical path. This phenomenon is accompanied by the propagation of an evanescent wave (EW), with the same wavelength of the incident beam and with intensity decaying exponentially within a characteristic distance of about 100–200 nm inside the second medium (i.e., the biofluid to be tested) (Figure 1).

The reduced penetration depth of the EW can be exploited to limit the excitation volume to a small portion of the sample, thus reducing the background contribution from deeper regions, while retaining a wide field configuration [6]. In this configuration, only molecules located within the EW region are excited, enhancing the contrast and improving the detection sensitivity down to the single molecule level [7]. Depending on the type of labels or probes linked to the analytes (fluorophores, metal particles), different optical signals can be detected, from fluorescence to scattering (both elastic and inelastic) [7, 8, 9]. The choice of TIR scattering microscopy, using metallic nanoparticles as probes, is encouraged by the characteristic NPs large scattering cross‐section. The high binding affinity between the gold nanoparticles (AuNPs) and the analytes immobilized in the EW region can be exploited to further enhance the scattered optical signal up to 6–8 orders of magnitude, enabling for the detection of single particles [10]. Moreover, to improve the specificity towards specific analytes attached to the coverslip in the EW region, the surface chemistry of AuNPs can be engineered to induce specific NP‐analytes capturing, thus enabling their selective recognition [11]. This strategy allows the detection of specific biomarkers in unprocessed biological matrices, thus avoiding pre‐processing procedures or separation steps that could alter the chemistry of the samples to be analyzed and extend the time for analysis. Furthermore, thanks to the recent technological advances in micro‐ and nanofabrication processes, EW biosensors could also strongly benefit from integration with lab‐on‐chip (LoC) microfluidic devices, thus enabling smart fluids handling, efficient mixing, sorting, or separation of a few microliters of samples, as well as implementation of multiplexing analysis [12, 13, 14].

Here we report on the full development of a modular, low‐cost bench‐top TIR detection system characterized by high portability to be coupled with a NPs‐based biosensor for the screening of analytes in biofluids, and on their further miniaturization within LoCs. The combination of microfluidic chambers containing engineered NPs for the selective recognition of specific biomarkers with the TIR scattering setup gives an advanced and versatile optical biosensor platform allowing for the incorporation of an almost limitless range of biorecognition probes, precisely and robustly conjugated to the sensor by covalent surface chemistry approaches. As a proof of concept, following previous works published by our group [15], the surface of NPs has been fully functionalized with ad‐hoc synthesized nonnatural antimicrobial peptide, M33, engineered to specifically recognize bacterial lipopolysaccharides (LPS). M33 is a tetra‐branched cationic amphipathic peptide, in which a lysine scaffold holds four identical sequences. This tetra‐branched form confers high resistance to protease and peptidase activities. The peptide is active against a panel of clinically important Gram‐negative bacteria [16], and it has been characterized in preclinical stages for efficacy (sepsis, lung infections, and skin infections), full toxicity, bio‐distribution, excretion, selection of resistances, gene toxicity, mechanism of action, immunomodulatory activity and time‐kill concentrations [17]. As many other cationic AMPs, M33 binds LPS [18, 19]. M33 was synthesized in a thiolated form, carrying a cysteine residue at the C‐terminus to be used as a linker for the AuNSps.

Increased levels of LPS concentration in blood indicate severe infections and sepsis. Thus, early and precise diagnosis with liquid biopsy could be crucial in improving patients' prognosis [20, 21, 22]. First, the peptide/NP ratio was optimized to achieve saturation of the gold surface, while ensuring the retaining of the colloidal stability at the same time. Then, a low‐cost bench‐top TIR microscope was successfully designed and realized. It exploits EW scattering to reveal analytes in fluids with sensitivity and specificity comparable to standard bulk TIR microscopes, but with a strong reduction of costs and dimensions. The proposed synergic combination of advanced EW‐based compact setup and ad‐hoc functionalized NPs for the screening of LPS in real serum samples will pave the way for possible applications in clinics for early‐stage diagnosis of sepsis, with an advanced level of automatization and high throughput.

## 2 Experimental

### 2.1 Implementation of the Portable Optical Detection System

**FIGURE 2:**
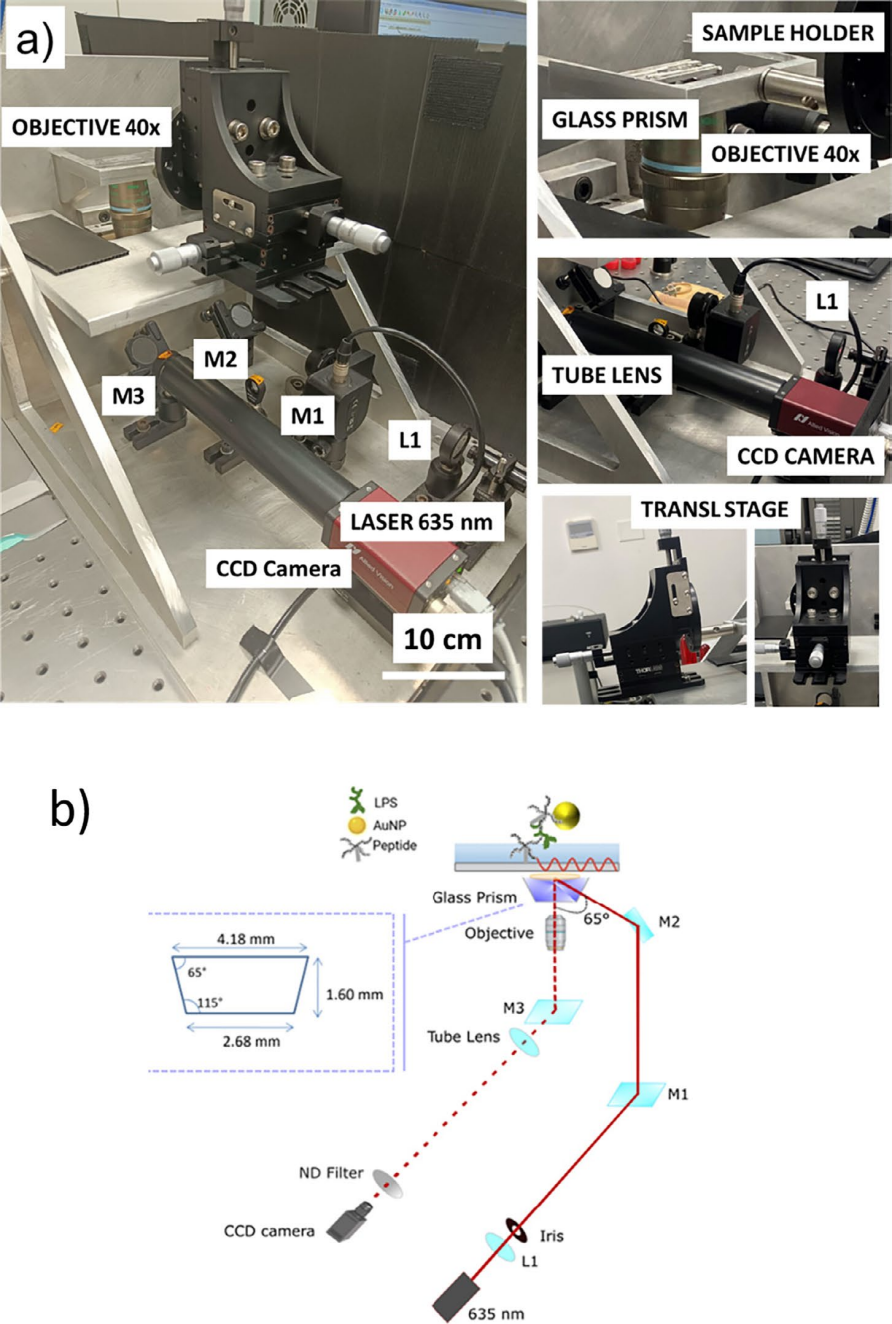
(a) Photos of the compact TIR setup from different views, showing the optomechanical components described in the main text. (b) Scheme of the optical path. L1: Collimating lens (*f* = 20 mm); M1, 2, 3: Dielectric mirrors. Inset: Geometry and dimensions of the glass microprism.

The optical system developed for the detection of the scattering signal coming from gold NPs is based on a glass micro‐prism to generate an EW through TIR. This prism‐based design makes the system extremely compact, portable, and low‐cost. At the same time, a key advantage of measuring nanoparticle scattering is the high sensitivity of the analysis. The experimental setup was designed to house all optical elements within a custom‐built metallic box, reducing its size to 40 × 30 × 40 cm^3^ and facilitating transport (Figure 2a). The optical scheme of the prism‐based TIR microscope, illustrated in Figure 2b, allows for significant size reduction compared to standard objective‐based TIR microscopy setups, as reported in Table 1. In particular, the illumination source consists of a 640 nm‐diode laser with 10 mW power, collimated by a 20‐mm lens (L1). Mirrors M1 and M2 direct the excitation beam towards an ad‐hoc glass micro‐prism before it reaches the sample holder. M1 raises the excitation beam perpendicularly, while M2, which is fixed on the vertical wall of the metallic box, adjusts the inclination of the laser beam via a micrometer screw to achieve the incidence critical angle required for TIR. The glass micro‐prism is optically coupled to the glass coverslip through immersion oil, which has the same refractive index of glass (*n*_glass_ = *n*_oil_ = 1.516). Then, an iris positioned along the laser's beam excitation path, in a plane conjugated to the image plane and the objective's focal plane, spatially limits the excitation beam and regulates the illumination area. During measurements, the LoC‐NPs sample is placed on a support atop the micro‐prism, and mounted on a 12.7 mm XYZ translation stage with standard micrometer screws, allowing for the spatial exploration of the sample and scanning measurements. After excitation, the scattered signal is collected by a CSI Plan Fluor 40× Nikon objective, which can be focused on the desired plane by moving the objective in the z‐direction using a micrometer screw. The objective, characterized by a 0.6 numerical aperture (NA) and an extra‐long working distance (2.8–3.6 mm), accommodates the distance constraints imposed by the glass micro‐ prism's dimensions (Figure 2b, left inset). The collected signal is then transmitted to a CCD camera through mirror M3 and a tube lens (*f* = 200 mm). The tube lens and the CCD camera are optically isolated from the environment by an 18 cm‐long black tube housing an appropriate ND filter (with OD varying from 0.5 to 2.0) to attenuate the scattering signal and prevent camera saturation. Finally, the CCD camera is connected to a lab computer for image acquisition and analysis, explained in detail in Section 2.3.

**TABLE 1:** detectable lowest concentration and technical characteristics for the traditional TIR bulky setup and the novel compact TIR prototype.

Feature	Traditional TIR setup	Compact TIR setup
Lowest conc det	1 ag/mL	1 fg/mL
Dimensions	Standard optical table	40 × 40 × 30 cm
Camera FOV	42 μm × 42 μm	221 μm × 166 μm
Camera type	EMCCD	CCD
Lateral resolution	346 nm	702 nm
Overall cost	~150 k €	~10 k €

### 2.2 Synthesis of Functionalized Nanoparticles for the Selective Recognition of LPS

Functionalized nano‐constructs were prepared starting with the synthesis of citrate‐stabilized gold nanospheres (AuNSps) of 15 nm of diameter using the Turkevich‐Frens synthetic protocol [23], further details are listed in Supporting Information: Section S1. Indeed, the 15 nm size provides a robust and reliable basis for achieving these objectives while avoiding the challenges associated with larger, more polydisperse nanoparticles.

**FIGURE 3:**
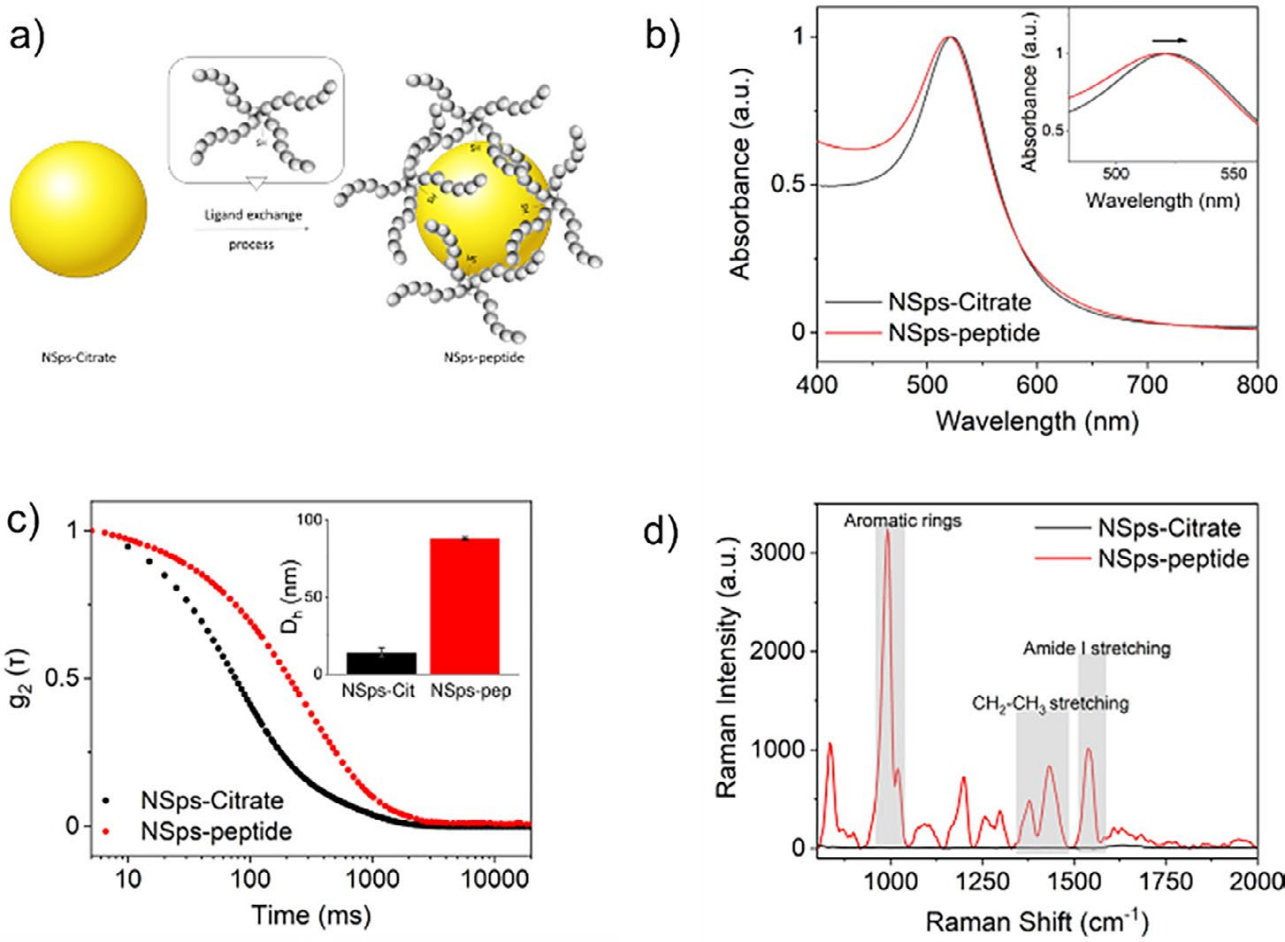
(a) Schematic procedure of gold nanoparticles functionalization with the thiol‐functionalized peptide synthesis for the selective capturing of LPS. Characterization of NPs‐peptide construct: (b) UV–Vis absorption spectra of NSps‐citrate (black line) and NSps‐peptide (red line). Inset shows the region 520–560 nm; (c) autocorrelation curves of NSps‐citrate (black line) and NSps‐peptide (red line) measured through Dynamic Light Scattering analysis. Inset shows size distribution and ζ potential of NSps‐citrate and NSps‐peptide; (d) Raman spectra of NSps‐citrate (black line) and NSps‐peptide (red line). Measurements were performed with a 785 nm laser, 20 mW, time acquisition 60s, and 2 accumulations.

An exchange ligand process was then implemented immediately after synthesis [24, 25, 26, 27], to functionalize the gold nano‐surface with thiolated‐peptide molecules designed to recognize bacterial lipopolysaccharides (LPS) changes in the serum of patients with severe infections or sepsis (Figure 3a). Peptide synthesis details are listed in section S1 of the SI. The plasmonic spectra of the colloidal solution exhibited a characteristic resonance peak at approximately 520 nm for citrate‐stabilized AuNSps, which shifted slightly to 522 nm upon peptide conjugation due to modification of the extinction coefficient (Figure 3b). Moreover, considering that the molecular weight of the peptide (5000 Da) is 25 times higher than citrate molecules (around 200 Da), peptide conjugation was also confirmed by changes in the size of AuNSps from 14 ± 3 to 88 ± 1 nm, as observed through autocorrelation curves and histograms in Figure 3c. Raman measurements further confirmed peptide conjugation to the AuNSps surface, with vibrational peaks indicative of amino acids becoming predominant due to the presence of the metallic nanostructures when the peptide is attached to the NPs surface (Figure 3d).

### 2.3 Imaging Processing and Analysis

**FIGURE 4:**
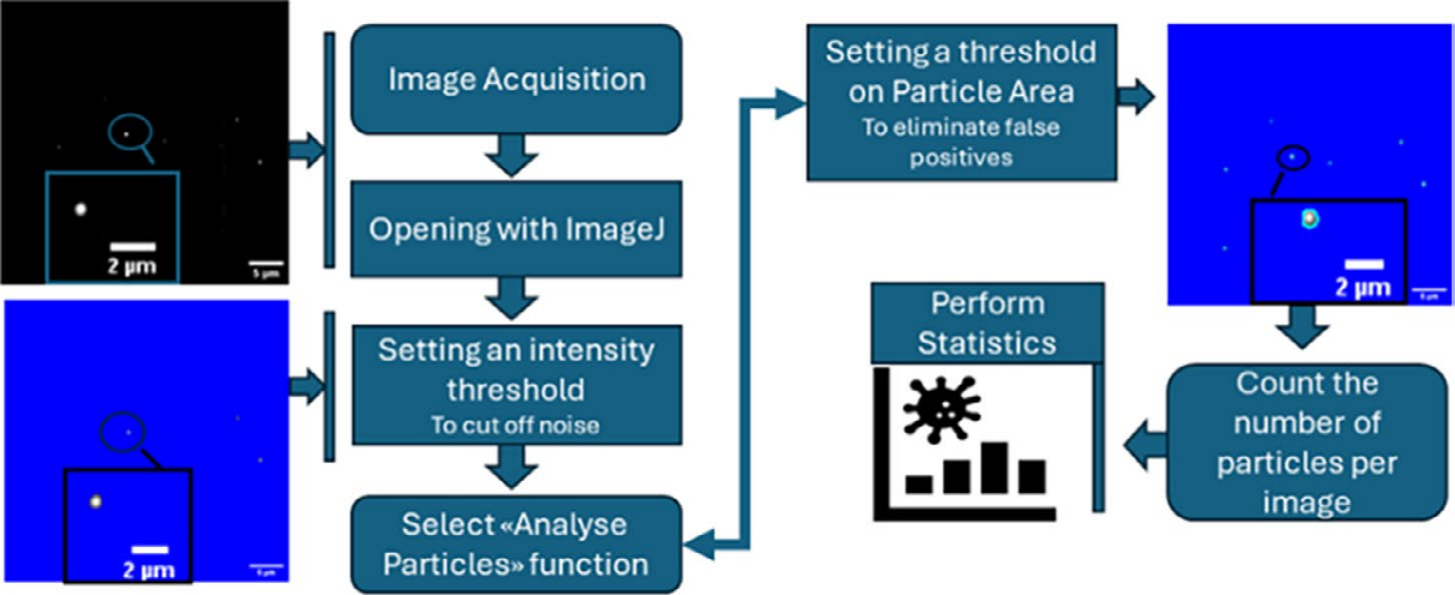
Graphical scheme of image processing procedure: First, the image is opened with ImageJ and a threshold value is chosen to remove the background noise. After noise subtraction, the function “Analyse particles” is run on data with a minimum size value for particles of 10 pixels^2^. This value comes from the calculation of the lateral resolution of the image, which is 702 nm (see Supporting Information: Section S7). From this analysis, the number of particles detected, their surface, and their average intensities are given, for each image.

The acquired images were analyzed and post‐processed through ImageJ by performing the procedure described in the scheme in Figure 4.

Detailed sample preparation procedures and experimental parameters are reported in Supporting Information: Sections S2 and S6. After image processing, statistical analysis of the obtained values was performed as follows:

- 20 images were collected at each LPS concentration and were processed as schematically described previously and in Figure 4.
- The AuNPs density d was obtained by dividing the number of AuNPs detected in each image by the image area (110 × 83 μm^2^).
- The average NPs density and its corresponding standard error were calculated from the density values distribution from (ii).
- Finally, to establish statistical differences between consecutive average densities of LPS, pair sample *t*‐tests were conducted.

## 3 Results and Discussion

### 3.1 Validation and Characterization of the Compact Setup on Synthetic Samples

The optical performances of the system, combining the compact EW‐optical reader with functionalized NPs, were tested by performing measurements of synthetic fluid samples with known concentrations of LPS in solution. Thanks to the functionalization of both the glass surface and the AuNPs with the peptide for selective LPS targeting, the attachment of a number of AuNPs in the EW region is expected in the presence of LPS in solution and self‐assemble in a “sandwich‐like” configuration as shown in Figure S1. By setting an appropriate AuNPs concentration (0.14 nM) to be kept fix over measurements of samples with increasing LPS concentration, a direct relation between the number of nanoparticles attached to the surface in the EW region and LPS concentration in solution is obtained. To achieve this result, different components of the “sandwich” must be added to the flow chamber in sequence as follows: first, the glass surface is saturated with the peptide (Figure S1a), then the solution to be probed with the specific LPS concentration is added and incubated for 4 min to let LSP molecules attach specifically to the peptide‐functionalized glass surface (Figure S1b). After a washing step to remove unbounded LPS molecules, a final incubation with a fixed concentration of peptide‐decorated AuNPs is performed (Figure S1c) for 4 min. Scattering images of samples at increasing LPS concentrations were taken and analyzed as described previously.

**FIGURE 5:**
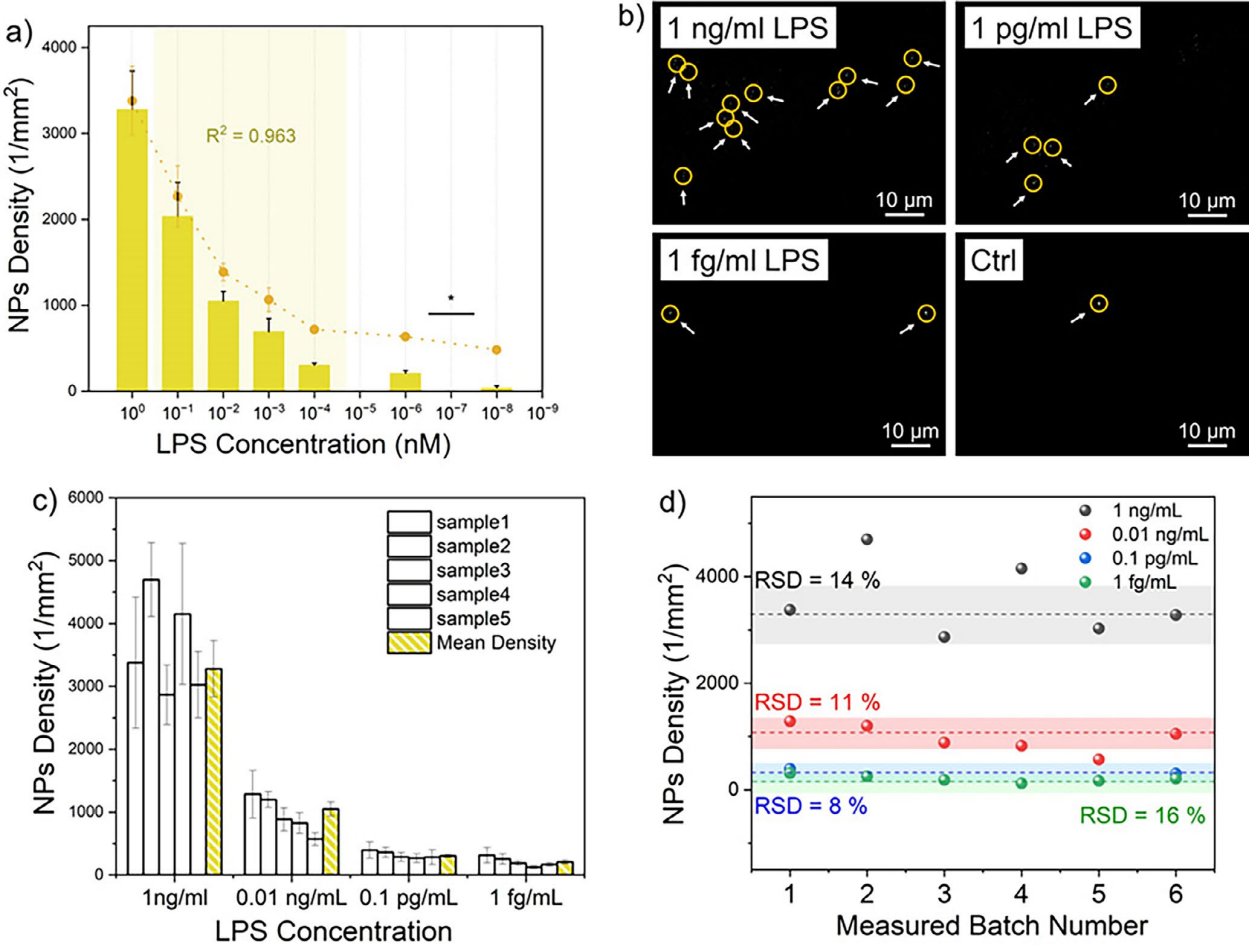
(a) Scattering measurements were performed at LPS concentrations spanning from 10^−6^ to 1 ng/mL. *N* ≥ 20 for each LPS concentration. Error bars indicate the standard error. NPs mean density in function of LPS concentration was graphed both in histograms and dot forms, and the shaded area shows the linear dynamic range. Two‐sample student t‐tests have been performed to evaluate the statistical differences between different LPS concentrations. The lowest concentration that can be detected with a statistically significant *p* value is in the femtogram per milliliter (fg/mL) range. (b) Scattering images at different LPS concentrations were acquired with the compact optical setup. Bright circled spots are scattered AuNPs. Scalebar 10 μm. (c) Histogram showing the reproducibility measurements performed on four different LPS concentrations. Five different LOC‐NPs samples were observed for each concentration (white bars), (*N* = 24 for each concentration) and the mean value of their density distribution was calculated, along with its standard error. (d) Batch‐to‐batch NPs density variation for the four tested concentrations.

The results were then compared with the performances of a TIRF, objective‐based bulky setup, described in detail in section “Validation of effective EW scattering of NPs and sensitivity establishment using the traditional bulky TIR Microscope” in Supporting Information: Sections S4 and S5 [28, 29, 30]. Peptides at different concentrations, ranging from 10^−6^ to 1 ng/mL, were dispersed in a PBS buffer as described in section “LOC‐NPs sample preparation” of Supporting Information: Section S3. The field of view (FOV) analyzed corresponds to 110 × 83 μm^2^, and *N* ≥ 20 FOVs were collected for each LPS concentration. Image analysis and statistics on data were performed as described in Section 2.3. As expected, experimental results are reported in Figure 5a, showing that the density of NPs increases with increasing LPS concentrations, thus demonstrating the capability of the system to distinguish diverse LPS concentration levels. Typical images acquired at different LPS concentrations are shown in Figure 5b. Data belonging to the linear dynamic range, highlighted with a yellow shaded area in the graph in Figure 5a, were used to build the calibration curve (Supporting Information: Section S9), correlating the NPs density with the LPS concentration. The limit of detection was considered to be 1 fg/mL, which is the lowest LPS concentration showing an average NPs density statistically different from the density of the control sample, where LPS are not present. This sensitivity is three orders of magnitude higher than the bulk setup (1 ag/mL), likely owing to the less‐performing optical components chosen to limit the cost and size of the system (Table 1). However, the setup demonstrated improved sensitivity compared to techniques currently used in clinics, such as commercial ELISA kits, which report limits of detection in the tens or hundreds of pg/mL range [31, 32].

Once the capability of the compact prototype to detect highly diluted analytes in aqueous solutions has been validated and the sensitivity of the system established, measurements were replicated in multiple samples to test reproducibility. To this end, four LPS concentrations in the range from 1 ng/mL to 1 fg/mL were chosen, five samples were prepared for each concentration, and images of 24 different FOVs were acquired for each sample. NPs density was measured for each FOV and a mean NPs density was calculated with the corresponding standard error. Afterward, the average mean density was calculated over five samples for each concentration to extract a final mean density with standard deviation to evaluate the reproducibility of the measurement procedure (in Figure 5c). Further analysis was done by calculating the relative standard deviation (RSD) of the NPs density for the four concentrations tested, as an indicator of the accuracy. RSDs of 14%, 11%, 8%, and 16%, respectively were calculated, thereby demonstrating good reproducibility (Figure 5d).

### 3.2 Validation of the Compact Setup on Human Samples and Accuracy Establishment

**FIGURE 6:**
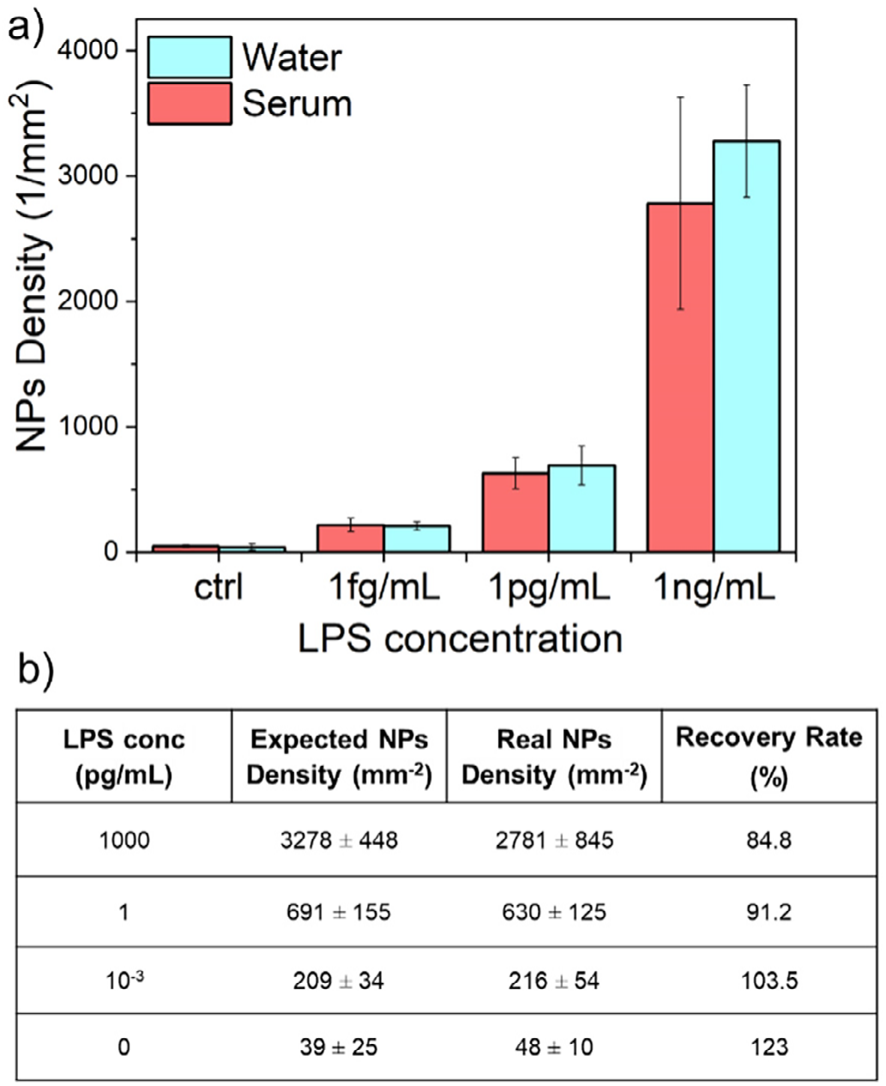
(a) Mean NPs density values obtained from experiments in human serum (red bars, *N* > 60) and in synthetic aqueous samples (blue bars, *N* > 20) at specified LPS concentrations. (b) Table comparing measured and expected mean densities at specific LPS concentrations and the corresponding calculated recovery rate (RR) %.

Finally, the capability of the compact setup was further tested on human serum samples enriched with known concentrations of LPS. Serum vials derived from healthy patients were provided by the Department of Medical Biotechnology of the University of Siena, and were used to perform measurements. Serum samples spiked with three different concentrations of LPS (1 ng/mL, 1 pg/mL, and 1 fg/mL) were measured. The setup successfully detected LPS concentrations down to 1 fg/mL. Expected NPs density values observed in Figure 5c were then compared with the actual values obtained from measurements in spiked serum, to quantify the recovery rates (RR) at each LPS concentration. RR was obtained by dividing the mean NPs density value measured in spiked serum samples by the expected density value. All values are listed in the table in Figure 6b. The graph in Figure 6a compares densities expected values (blue bars) and densities obtained from measurements (red bars) at each LPS concentration. Comparison with measurements in aqueous samples showed no significant difference, thus demonstrating the capability of the system to probe human biofluids effectively.

## 4 Conclusion

In conclusion, our study shows the advantages of combining confined EW excitation, with highly efficient scatterers such as gold nanoparticles, as a robust methodology for highly sensitive detection of target analytes in liquid samples. The implementation of this approach with a compact and portable TIR microscope further demonstrates its potential for the clinical scenario. Our investigation thoroughly evaluated the limit of detection and reproducibility of this compact setup, characterized by reduced dimensions and constructed using cost‐effective optomechanical components. Notably, this easily reproducible system exhibits the capability for rapid, accurate, and multiplexed analysis. As a proof of concept, we applied the device to the detection of bacterial lipopolysaccharides (LPS) in human serum, a key indicator of potential sepsis. The achieved sensitivity in the range of fg/mL LPS concentration stands as a remarkable accomplishment, especially when considering the limited cost of the system and its competitive performance against established techniques. Given the versatility of nanoparticle (NP) engineering, our proposed device emerges as a versatile tool for the real‐time analysis of various diseases in human biofluids. Its compact design, ease of use, and affordability make it a promising candidate for widespread application in clinical settings. The successful validation on human serum samples from patients, coupled with its ability to detect very low concentrations of target analytes, pave the way for its potential application in mass population screening, early‐stage disease diagnosis, and bed monitoring.

## Author Contributions

The manuscript was written through contributions of all authors. All authors have approved the final version of the manuscript.

## Conflicts of Interest

The authors declare no conflicts of interest.

## Supporting information


**Data S1.** Supporting Information.

## Data Availability

The data that support the findings of this study are available from the corresponding author upon reasonable request.
